# Circadian Syndrome Is Associated with Dietary Patterns among Middle-Older Americans: The Health and Retirement Study

**DOI:** 10.3390/nu16060760

**Published:** 2024-03-07

**Authors:** Abeer Ali Aljahdali, Zumin Shi

**Affiliations:** 1 Department of Clinical Nutrition, Faculty of Applied Medical Sciences, King Abdulaziz University, Jeddah 21589, Saudi Arabia; aaoaljahdali1@kau.edu.sa; 2 Department of Nutritional Sciences, University of Michigan, Ann Arbor, MI 48109, USA; 3 Human Nutrition Department, College of Health Sciences, QU Health, Qatar University, Doha P.O. Box 2713, Qatar

**Keywords:** diet, dietary patterns, circadian rhythms, circadian syndrome, Health and Retirement Study, middle-older adults, US

## Abstract

Population aging is a global demographic characteristic of the 21st century, and healthy eating is a core component of healthy aging. However, limited evidence is available among older adults for associations between diet quality and circadian syndrome (CircS). Thus, this study examined associations between dietary patterns and CircS among a representative sample of middle-older adults in the US. The sample comprised middle-older adults enrolled in the 2016 core wave of the Health and Retirement Study (HRS) and one of its sub-studies, the 2013 Health Care and Nutrition Study (HCNS). A food frequency questionnaire was used to quantify habitual food intake and identify dietary patterns using a factor analysis. CircS was defined based on the existence of ≥4 components of metabolic syndrome and indicators of sleep disorders and depression. A total of 4253 middle-older adults with a mean age (SD) of 65.4 (10.0) years were included in the study. The prevalence of CircS was 35.9%. Comparing extreme quartiles of the “Prudent Pattern”, the odds ratio (95% CI) for CircS was 0.72 (0.55–0.94), and it was 1.47 (1.10–1.95) for the “Western Pattern”. The “Western Pattern” was positively associated while the “Prudent Pattern” was inversely associated with the odds of CircS among middle-older adults.

## 1. Introduction

Metabolic syndrome (MetS) is a cluster of physiological abnormalities, including central obesity, impaired glucose metabolism, elevated blood pressure, and dyslipidemia [1], that have been identified as risk factors for incident cardiovascular diseases (CVDs), all-cause mortality, cardiovascular mortality [2,3], and other chronic diseases [4,5]. Globally, CVDs were the leading causes of death and significant contributors to higher health system costs in 2021 [6]. In recent years, the concept of MetS has been expanded to incorporate evidence showing the potential role of circadian rhythm disruption in CVDs [7,8,9] and has been labeled circadian syndrome (CircS) [10]. CircS encompasses all components of MetS plus indicators of depression and sleep disorders [10]. Previous studies have shown that the magnitude of associations between CircS and the prevalence and incidence of CVDs was higher than the magnitude of associations between MetS and CVDs [11,12]. Also, adults with CircS had higher odds of testosterone deficiency [13], stroke [14], and kidney stones [15] compared to counterparts who did not have CircS.

Diet is a well-known leading, modifiable risk factor for CVDs, and unhealthy eating behaviors were associated with 6.58 million incidences of CVD-related mortality [6]. Furthermore, a meta-analysis of forty observational studies showed that prudent/healthy patterns were associated with a reduced MetS risk and meat/Western/unhealthy patterns were associated with an increased MetS risk [16]. However, there is a dearth of knowledge on the association between dietary intake and CircS. To the best of the authors’ knowledge, only one study examined the association between eating patterns and CircS using a large, nationally representative sample of US adults aged 20–60+ years enrolled in the National Health and Nutrition Examination Survey (NHANES); the authors found that the consumption of refined grains, fats, sugars, and red meats—the Western pattern—was associated with higher odds of having CircS by 1.96 times (95% CI 1.53–2.53), while the consumption of plant-based food and whole grains—the prudent pattern—was associated with lower odds of having CircS by 0.71 times (95% CI 0.58–0.86) [17]. Further observational studies are warranted to confirm the association between diet and CircS to validate the findings on the association between dietary patterns and CircS.

The population of older adults is growing, causing population aging to be considered a dominant and global demographic characteristic of the 21st century [18]. In the USA, a documented increase in the percentage of the older adult population during the last decade was reported [19,20]. In 1920, adults aged ≥65 years accounted for 4.7% of the total US population (1 in every 20 people), but they accounted for 16.8% (1 in every 6 people) in 2020 [21]. Furthermore, it is expected that in 2034, the number of older adults will exceed the number of children <18 years [19]. Aging is a natural process that is characterized by gradual changes in physiological processes that could contribute to increases in morbidity and mortality [22]. Healthy eating is a core component of healthy aging [23,24]. However, previous studies showed lower diet quality among older adults aged >50 years enrolled in the Health and Retirement Study (HRS) [25,26,27]. Furthermore, a reduction in diet quality among older adults aged ≥65 years was reported between 2001 and 2018 based on data from NHANES [28]; the mean primary American Heart Association score decreased from 19.84 (95% CI 19.40–20.29) in 2001 to 18.28 (95% CI 17.84–18.73) in 2018 [28]. Therefore, special attention should be paid to diet quality among older adults and how it associates with health outcomes; such evidence will provide insights into public health intervention measures to reduce the burden of diseases on older adults by improving their diet quality. It is well known that diet quality is associated with MetS among adults in the US [29].

To the best of our knowledge, there are no previous studies that have investigated the association between diet quality and CircS among a nationally representative sample of middle-older adults in the US. Therefore, the current study was conducted to examine the association between diet quality and CircS by analyzing data from the HRS.

## 2. Materials and Methods

The HRS, initiated in 1992, is a biennial national panel survey focusing on middle-older Americans [30]. It is funded by the National Institute on Aging (NIA) and the Social Security Administration. As part of the HRS, the 2013 Health Care and Nutrition Study (HCNS) collected information about food consumption using a validated 164-food-item food-frequency questionnaire (FFQ) [31]. In total, 8035 participants took part in the HCNS [32]. The participants’ sociodemographic, lifestyle, and health-related data were based on the HRS 2012 and the 2016 RAND HRS data file (Version P). The RAND HRS data file is a user-friendly file created by the RAND Center for the Study of Aging. It is derived from all waves of the HRS and contains cleaned and processed variables. Anthropometric measurements were based on two waves of the HRS (2012 and 2016) as only half of the participants took the measures in each wave. Biomarker data, encompassing total cholesterol (TC), high-density lipoprotein cholesterol (HDL-C), triglycerides (TGs), and hemoglobin A1C (HbA1C), were derived from the 2016 survey.

In the current study, out of the 8035 individuals enrolled in the HCNS, we excluded those aged below 50 years in the HCNS survey or those who had a survey weight of zero in the first step of sample selection (n = 28). We then excluded respondents who did not have information on MetS (n = 3647) or had daily energy intakes falling outside the commonly used allowable range of 600–6000 kcal/d for men and 500–5000 kcal/d for women (n = 107). A person needed to have information available for at least three components of MetS to be included in the analysis.

### 2.1. Outcome Measure

#### Circadian Syndrome

CircS was defined based on seven components including the presence of (1) an increased waist circumference (≥102 cm in males, ≥88 cm in females); (2) elevated blood pressure (systolic ≥ 130 and/or diastolic ≥ 85 mm Hg) or drug treatment for hypertension; (3) low HDL-cholesterol (<40 mg/dl in males and <50 mg/dl in females) or drug treatment for dyslipidemia; (4) high TG (≥150 mg/dL) or drug treatment for dyslipidemia; (5) elevated fasting glucose (≥100 mg/dL, drug treatment of elevated glucose is an alternate indicator), (6) a sleep disorder (sleep duration < 6 h/day or >9 h/day or a doctor-diagnosed sleep disorder), and (7) depression. Participants with four or more components were defined as having CircS.

Depression was measured using the eight-item Center for Epidemiologic Studies Depression Scale (CES-D) [33]. A participant with a CES-D score of ≥4 was defined as having depression symptoms. MetS was defined using revised criteria derived from various international organizations, including the International Diabetes Federation Task Force on Epidemiology and Prevention [1].

### 2.2. Exposure Measure

#### Dietary Patterns

Food consumption data obtained from the Food Frequency Questionnaire (FFQ) were initially condensed into 35 food groups categorized based on similarities in nutrient profiles and cooking methods [27]. Utilizing the principal component method, a factor analysis was applied to formulate dietary patterns, employing the intake information of these 35 food groups as input variables. The determination of the number of dietary patterns adhered to specific criteria: (1) eigenvalues exceeding 1.0; (2) scrutiny of a scree plot, and (3) ensuring factor interpretability. Factors were labeled based on our interpretation of pattern structures and the food items displaying the highest factor loadings. Factor loadings, indicative of correlations between food items and identified factors, were pivotal in this process. Factor scores were computed as the sum of the standardized intake product for each food item and its respective factor loading. Supplemental Table S1 shows the factor loadings of two dietary patterns.

### 2.3. Covariates

Various self-reported factors were treated as covariates in the study, including age, gender, racial background (categorized as White, Black African American, or other), education (grouped into less than high school, high school graduate or equivalent, and college graduate or above), frequency of vigorous physical activity per week (classified as <2 times/week or ≥2 times/week), smoking status (participants were categorized as non-smokers, ex-smokers, or current smokers), and alcohol consumption (indicated by yes or no).

### 2.4. Statistical Analyses

In the analysis, dietary pattern scores were recoded into quartiles (from low to high). To assess group differences for categorical variables, the chi-squared test was applied, while continuous variables were compared using an ANOVA. The multivariable model incorporated survey weights to address intricate multistage probability sampling. Logistic regression was employed to examine the associations between dietary patterns and CircS. Three models were utilized: model 1 remained unadjusted, model 2 was adjusted for age, sex, and energy intake, and model 3 was further adjusted for race, education, smoking, alcohol consumption, and physical activity. Testing for a linear trend involved using the original dietary pattern scores as continuous variables in the regression model. Subgroup analyses were conducted to test for multiplicative interactions between dietary patterns and sex, age (<65 or ≥65), race, education, vigorous activity, smoking, and alcohol consumption. The final analytic sample was 4253. In the analytical sample, 2329 participants had missing values for central obesity. For sensitivity, we replaced the missing central obesity values with obesity values based on the body mass index (BMI). As the main findings remained, we did not report the sensitivity analysis results.

All the analyses were performed using STATA 18.0 (Stata Corporation, College Station, TX, USA). Survey weight was used in all the multivariable models. Statistical significance was considered when *p* < 0.05 (two-sided).

## 3. Results

Table 1 shows the overall and sex-stratified descriptive characteristics of the analytical sample. The overall mean age of the participants was 65.4 (SD 10.0) years. Female participants accounted for approximately 60% of the analytical sample, and 78% of the study population were White Americans. The unweighted prevalences of MetS and CircS were 48.3% and 35.9%, respectively (Table 1). The weighted prevalences of MetS and CircS were 46.0% and 34.3%, respectively.

Two dietary patterns were identified. The first pattern was named the “Prudent Pattern” as it had high loadings for different types of vegetables, fruit, seafood, olive oil, and nuts. The “Western Pattern” was loaded heavily with processed meat, red meat, refined grains, French fries, condiments, snacks, and soft drinks. The two dietary patterns explained 14.0% and 8.6% of the variance in food intake, respectively (Supplementary Figure S1).

The distribution of sociodemographic and lifestyle characteristics across quartiles of the “Prudent and Western Patterns”, respectively are presented in Supplementary Tables S1 and S2. The “Prudent Pattern” was positively associated with the level of education, while the “Western Pattern” was inversely associated. Participants with high levels of consumption following the “Prudent Pattern” were more likely to undertake vigorous activity and less likely to smoke. However, the opposite was true for the “Western Pattern”. Men were more likely to have a high intake of food groups in the “Western Pattern”, while women were more likely to have a high intake of food groups in the “Prudent Pattern” (Supplementary Tables S1 and S2).

Associations between the “Western and Prudent Patterns” and CircS are shown in Table 2. Model 2 showed that the “Prudent Pattern” was inversely associated with CircS. Furthermore, one unit/SD increase in the “Prudent Pattern” score was associated with a 15% reduction in the odds of having CircS (OR = 0.85, 95% CI 0.76–0.95). However, the association was attenuated and became statistically not significant after further adjusting for smoking, alcohol consumption, and physical activity (Model 3). For the “Western Pattern”, a positive association was detected in Model 2 (*p*-trend < 0.001). Middle-older adults in the fourth quartile of the “Western Pattern” were 1.47 times (95% CI 1.10–1.95) more likely to have CircS compared to middle-older adults in the reference group in the fully adjusted model (Model 3). Also, one unit/SD increase in the “Western Pattern” score was associated with a 29% increase in the odds of having CircS (OR = 1.29, 95% CI 1.16–1.44) (Table 2).

**Figure 1:**
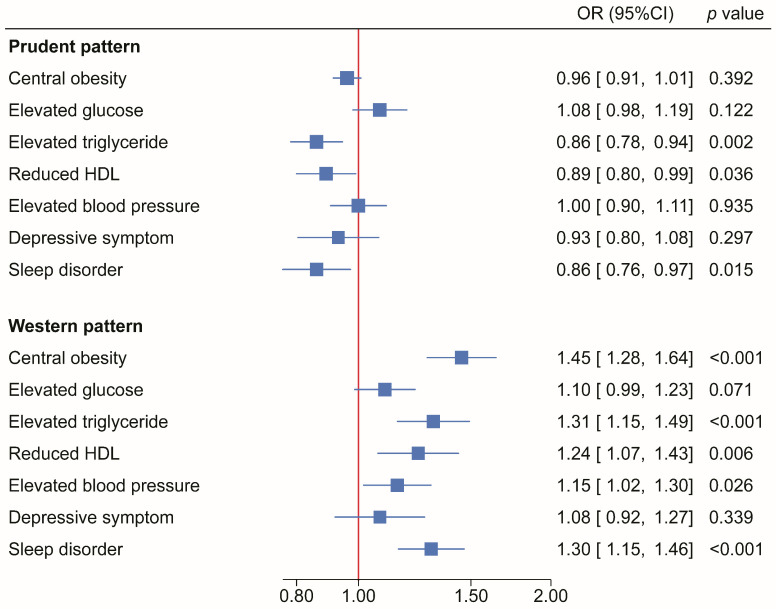
Associations (odds ratios (95% CI) between dietary patterns and components of circadian syndrome among adults participating in Health and Retirement Study. *p*-values were calculated using dietary pattern score as a continuous variable in multivariable models. Models adjusted for age, sex, race, energy intake, physical activity, education, smoking and alcohol drinking.

Associations between scores for the “Western and Prudent Patterns” and the components of CircS are shown in Figure 1. The “Prudent Pattern” was inversely associated with elevated TG (OR= 0.86, 95% 0.78–0.94), reduced HDL-C (OR = 0.89, 95% CI 0.80–0.99), and sleep disorder (OR = 0.86, 95% CI 0.76–0.97). The “Western Pattern” was positively associated with central obesity (OR= 1.45, 95% CI 1.28–1.64), elevated TG (OR = 1.31, 95% CI 1.15–1.49), reduced HDL-C (OR = 1.24, 95% CI 1.07–1.43), elevated blood pressure (OR = 1.15, 95% CI 1.02–1.30), and sleep disorder (OR = 1.30, 95% CI 1.16–1.47) (Figure 1).

Associations between scores for the “Prudent and Western Patterns” and CircS stratified by some sociodemographic factors are shown in Table 3 and Table 4, respectively. There were no significant interactions between dietary patterns with most sociodemographic factors in relation to CircS. However, a positive association between the “Western Pattern” and CircS was only observed among adults aged ≥65 years. A borderline significant interaction between the “Western Pattern” and sex was found. The positive association between the “Western Pattern” and CircS was significant only in women but not in men. There was a borderline significant interaction between smoking and the “Western Pattern”. The “Western Pattern” was positively associated with CircS among non-smokers but inversely associated with CircS among current smokers.

## 4. Discussion

Using a nationally representative sample of older adults in the US, we found that in 2016, the prevalence of CircS was 29%. Furthermore, the “Western and Prudent Patterns”, the two patterns that represented the dietary intake among the study population, were collectively described as 22.6% of the variance in the reported intake. It was shown that the “Western Pattern” scores, characterized by a high intake of processed meat, red meat, refined grains, French fries, condiments, snacks, and soft drinks, were associated with a higher prevalence of CircS, while the “Prudent Pattern” scores, characterized by a high intake of vegetables, fruit, vegetables, seafood, olive oil, and nuts, were associated with a lower likelihood of CircS. In addition, the association between the “Western Pattern” and CircS was higher among men, subjects aged ≥65 years, those with higher levels of education, the White race, those engaging in low levels of physical activity, non-smokers, and those who did not consume alcohol. However, there was no evidence of any significant interaction effect in the subgroup analyses.

In the current study, we reported that the prevalence of MetS was higher than the prevalence of CircS. Our observation was consistent with previous studies that quantified the prevalences of the two syndromes [11,12,34]. As an illustration, using a nationally representative sample of adults aged >40 years enrolled in the China Health and Retirement Longitudinal Study, it was reported that the prevalence of CircS (30.2 [34] and 39.0% [11]) was lower than the prevalence of MetS (38.4% [34] and 44.7% [11]). Furthermore, among US adults aged ≥20 years enrolled in NHANES waves between 2005 and 2016, it was shown that the prevalence of CircS was 40.8% but 48.0% for MetS [12]; however, approximately 40% of participants had both CircS and MetS [12]. Despite the slightly lower prevalence of CircS than MetS, previous studies concluded that CircS provided a better prediction for CVDs than MetS [11,12]. Further studies are warranted to investigate the longitudinal association between CircS and health outcomes and examine the role of lifestyle factors such as diet, physical activity, and sleep in mitigating the association between CircS and health outcomes.

We found a positive association between the “Western Pattern” and CircS and an inverse association between the “Prudent Pattern” and CircS among older adults. Our findings are consistent with the reported inverse and positive associations for “Prudent/Healthy Patterns” and “Western/Unhealthy Patterns”, respectively, with MetS and its components [16,35,36,37,38,39,40], CVDs [41,42], sleep [43], and depression [44]. A meta-analysis distilled evidence from forty observational studies and concluded that the “Healthy Pattern” was associated with lower odds of MetS (OR = 0.85, 95% CI 0.79–0.91), while the “Meat/Western Pattern” was associated with higher odds of MetS (OR = 1.19, 95% CI 1.09–1.29) [16]. Furthermore, our data revealed that the “Western Pattern” was associated with higher odds of all CircS components except for higher blood glucose and depression; however, the “Prudent Pattern” scores were associated only with high TG, low HDL-C, and short sleep. As a result, our analysis showed that dietary pattern might exert different mechanisms in explaining the biological mechanism linking diet quality and CircS; thus, additional studies are needed to fully assess individual food groups concerning the CircS to gain more insights into the impact of nutrients or food groups, such as melatonin-rich food, on CircS.

It is well known that the circadian rhythm has a significant role in regulating metabolism and health [45,46], including but not limited to energy expenditure, appetite [47], and the cardiovascular system [48]. Furthermore, previous studies showed that a disruption in the circadian rhythm was associated with impaired metabolism, manifesting as impaired components of cardiometabolic health outcomes [8,49,50,51,52,53,54,55]. As a result, the inclusion of indicators of the desynchronization of circadian rhythm—depression and the night shift—as proposed additional components of the old definition of MetS is well supported [10]. Earlier evidence suggested the role of circadian rhythm disruption in the pathology of MetS [47,54,56,57,58] before the introduction of CircS [10]. It is worth noting that previous studies found a strong link between sleep disorders, inflammation, and insulin resistance, which all contribute to CVDs [59]. In addition, other studies identified the potential of a disrupted circadian system in the etiology of type 2 diabetes [55] and a strong link between MetS and depression [60,61,62,63]. We recommend that future studies use the concept of CircS instead of MetS to incorporate the well-identified role of the circadian rhythm.

Furthermore, the association between circadian rhythms and diet quality was examined in earlier studies; however, the exact biological mechanisms have not been elucidated. Evidence based on an experimental animal study showed that the disruption of the circadian rhythm and a high-fat diet were associated with impaired glucose homeostasis, and these two factors collectively were associated with an increase in body mass, an observation not noted among mice with only disrupted circadian rhythms and no low-fat diet [64]. Adverse metabolic consequences among shift workers were documented due to alterations to eating and sleeping behaviors [47]; nevertheless, social jetlag and eating jetlag could also contribute to negative cardiometabolic outcomes [47]. Moreover, it was proposed that desynchronized circadian rhythms were linked to changes in the gut microbat and that diet quality and meal timing play vital roles in the balance between circadian rhythms and the gut microbiota [65]. It was reported that mealtime is a stronger factor influencing the synchronization of the metabolism’s circadian rhythms [66]. Further studies are needed to examine the role of dietary intervention in mitigating the cardioembolic consequences of circadian disruption on human health.

This study has a few limitations worth discussing. We acknowledge that the use of a principal component analysis might be affected by the multiple subjective decisions made throughout the process of contrasting food groups. Nevertheless, subjectivity is a well-documented inherent limitation of this method [67], and the principal component analysis is considered one of the two types of dietary pattern analysis in epidemiological studies—an a *posteriori* approach [68]. Also, our dietary patterns collectively explained less than a quarter of the variance in dietary intake among the study population; however, we followed the same data reduction techniques used in a previous publication among HRS participants [27]. Moreover, despite the adjustment of multiple covariates, residual confounding cannot be ruled out. Also, we acknowledge the possibility of reverse causation.

## 5. Conclusions

In conclusion, a positive association was detected between CircS and the “Western Pattern” that is characterized by a high intake of processed meat, red meat, refined grains, French fries, condiments, snacks, and soft drinks. Conversely, an inverse association was reported among those who had higher scores for the “Prudent Pattern” characterized by a high intake of vegetables, fruit, seafood, olive oil, and nuts. There was no evidence of any significant interaction effect in the subgroup analyses. Given the use of a nationally representative sample of older adults, our findings are generalizable to older adults in the US, providing evidence on the importance of diet quality as a potential modifying factor for CircS among older adults. Thus, our study emphasizes promoting a healthy diet to reduce the burden of diseases among older adults in the US. We call not only for additional studies to explore longitudinal repeated assessments of dietary intake among older adults but also for studies to evaluate public health programs aimed at improving the accessibility and affordability of healthy food for older adults.

## Figures and Tables

**Table 1:** Sample characteristics among adults participating in the Health and Retirement Study.

	Total	Men	Women	*p*-Value
	n = 4253	n = 1736	n = 2517	
Age, (years)	65.4 (10.0)	66.0 (9.6)	65.0 (10.2)	0.001
Sex				<0.001
Men	1736 (40.8%)	1736 (100.0%)	0 (0.0%)	
Women	2517 (59.2%)	0 (0.0%)	2517 (100.0%)	
Race				0.008
White	3286 (77.5%)	1379 (79.8%)	1907 (75.9%)	
Black African American	632 (14.9%)	225 (13.0%)	407 (16.2%)	
Other	323 (7.6%)	124 (7.2%)	199 (7.9%)	
Education				<0.001
High school graduate or below	2073 (48.8%)	816 (47.0%)	1257 (50.0%)	
Some college/college graduate	1085 (25.5%)	393 (22.7%)	692 (27.5%)	
Post-college	1092 (25.7%)	526 (30.3%)	566 (22.5%)	
Smoking				<0.001
None	1934 (45.7%)	613 (35.5%)	1321 (52.7%)	
Ex-smoker	1889 (44.6%)	932 (53.9%)	957 (38.2%)	
Current smoker	410 (9.7%)	183 (10.6%)	227 (9.1%)	
Alcohol consumption				<0.001
No	1838 (43.3%)	632 (36.4%)	1206 (48.0%)	
Yes	2411 (56.7%)	1102 (63.6%)	1309 (52.0%)	
Vigorous physical activity				<0.001
<2 times/week	3183 (75.2%)	1219 (70.5%)	1964 (78.5%)	
≥2 times/week	1047 (24.8%)	510 (29.5%)	537 (21.5%)	
Central obesity	2849 (71.6%)	1065 (65.0%)	1784 (76.3%)	<0.001
Elevated glucose	2129 (50.3%)	940 (54.4%)	1189 (47.4%)	<0.001
Elevated	1504 (35.4%)	642 (37.0%)	862 (34.2%)	0.067
Reduced high-density lipoprotein cholesterol	1059 (24.9%)	413 (23.8%)	646 (25.7%)	0.16
Elevated blood pressure	2966 (69.8%)	1231 (70.9%)	1735 (69.0%)	0.17
Depression	543 (12.8%)	175 (10.1%)	368 (14.6%)	<0.001
Sleep disorder	1345 (31.6%)	602 (34.7%)	743 (29.6%)	<0.001
Metabolic syndrome	2055 (48.3%)	841 (48.4%)	1214 (48.2%)	0.89
Circadian syndrome	1527 (35.9%)	619 (35.7%)	908 (36.1%)	0.78
Energy intake (kcal/day)	1830.0 (793.5)	1932.3 (823.9)	1759.4 (764.0)	<0.001
Protein intake (g/day)	71.9 (33.2)	74.4 (33.6)	70.2 (32.9)	<0.001
Fat intake (g/day)	67.5 (32.1)	70.9 (33.7)	65.2 (30.8)	<0.001
Carbohydrate intake (g/day)	229.7 (109.0)	239.2 (109.6)	223.1 (108.0)	<0.001
Western dietary pattern	0.00 (1.00)	−0.09 (0.96)	0.06 (1.02)	<0.001
Prudent dietary pattern	−0.00 (1.00)	0.19 (1.05)	−0.13 (0.94)	<0.001

Data are presented as mean (SD) values for continuous measures and n (%) for categorical measures.

**Table 2:** Odds ratios (95% CI) for circadian syndrome by quartiles of dietary patterns among adults participating in the Health and Retirement Study.

	Quartiles of Intake					Intake as a Continuous Variable
	Q1 (Low)	Q2	Q3	Q4 (High)	*p*-Value *	
Prudent Pattern						
Model 1	1.00	0.76 (0.59–0.98)	0.71 (0.54–0.93)	0.64 (0.51–0.81)	0.004	0.88 (0.80–0.96)
Model 2	1.00	0.80 (0.62–1.04)	0.75 (0.56–1.00)	0.61 (0.46–0.81)	0.005	0.85 (0.76–0.95)
Model 3	1.00	0.83 (0.65–1.06)	0.85 (0.64–1.14)	0.72 (0.55–0.94)	0.086	0.91 (0.82–1.01)
Western Pattern						
Model 1	1.00	0.99 (0.78–1.27)	1.15 (0.90–1.46)	1.46 (1.16–1.83)	<0.001	1.21 (1.12–1.31)
Model 2	1.00	1.08 (0.83–1.41)	1.33 (1.05–1.69)	1.84 (1.41–2.40)	<0.001	1.42 (1.27–1.59)
Model 3	1.00	1.01 (0.78–1.30)	1.23 (0.96–1.58)	1.47 (1.10–1.95)	<0.001	1.29 (1.16–1.44)

* *p*-values were calculated using the dietary pattern score as a continuous variable in multivariable models. Model 1: unadjusted. Model 2: adjusted for age, sex, energy intake, race and education. Model 3: further adjusted for smoking, alcohol consumption, and physical activity.

**Table 3:** Subgroup analyses of the association between prudent dietary patterns and circadian syndrome among adults participating in the Health and Retirement Study.

	Prudent Pattern Patterns					
	Q1	Q2	Q3	Q4	*p* for Trend	*p* for Interaction
**Sex**						0.216
Men	1.00	0.88 (0.63–1.23)	0.97 (0.64–1.45)	0.96 (0.62–1.51)	0.926	
Women	1.00	0.75 (0.54–1.05)	0.74 (0.52–1.06)	0.58 (0.42–0.80)	0.004	
**Age**						0.478
<65	1.00	0.90 (0.63–1.28)	0.79 (0.50–1.25)	0.71 (0.47–1.09)	0.111	
≥65	1.00	0.80 (0.62–1.03)	1.00 (0.74–1.34)	0.84 (0.58–1.22)	0.640	
**Race**						0.008
White	1.00	0.83 (0.64–1.09)	0.85 (0.62–1.16)	0.75 (0.56–1.02)	0.090	
Black African American	1.00	1.28 (0.70–2.33)	1.91 (0.86–4.21)	0.62 (0.28–1.40)	0.915	
Other	1.00	0.36 (0.13–0.97)	0.34 (0.11–1.09)	0.65 (0.25–1.68)	0.367	
**Education**						0.695
High school graduate or below	1.00	0.87 (0.63–1.19)	1.00 (0.68–1.46)	0.99 (0.71–1.39)	0.954	
Some college/college graduate	1.00	0.88 (0.55–1.39)	0.87 (0.53–1.45)	0.68 (0.42–1.10)	0.131	
Post-college	1.00	0.64 (0.40–1.04)	0.61 (0.38–0.98)	0.48 (0.30–0.77)	0.005	
**Vigorous physical activity**						0.172
<2 times/week	1.00	0.78 (0.61–0.99)	0.81 (0.61–1.09)	0.62 (0.47–0.81)	0.004	
≥2 times/week	1.00	1.31 (0.63–2.72)	1.46 (0.72–2.93)	1.76 (0.83–3.77)	0.106	
**Smoking**						0.721
None	1.00	0.79 (0.56–1.11)	0.87 (0.50–1.49)	0.68 (0.42–1.11)	0.225	
Ex-smoker	1.00	0.77 (0.53–1.11)	0.80 (0.53–1.21)	0.72 (0.45–1.14)	0.185	
Current smoker	1.00	1.26 (0.65–2.47)	0.93 (0.51–1.73)	1.21 (0.40–3.60)	0.857	
**Alcohol consumption**						0.760
No	1.00	0.80 (0.58–1.10)	0.82 (0.55–1.22)	0.69 (0.47–1.01)	0.073	
Yes	1.00	0.88 (0.63–1.24)	0.92 (0.61–1.37)	0.81 (0.51–1.28)	0.452	

Models adjusted for age, sex, race, education, energy intake, smoking, alcohol consumption, and physical activity. Stratification variables were not adjusted in the corresponding models.

**Table 4:** Subgroup analyses of the associations between Western dietary patterns and circadian syndrome among adults participating in the Health and Retirement Study.

	Western Diet Pattern Patterns					
	Q1	Q2	Q3	Q4	*p* for Trend	*p* for Interaction
**Sex**						0.059
Men	1.00	1.02 (0.72–1.43)	1.12 (0.75–1.67)	1.11 (0.72–1.71)	0.574	
Women	1.00	0.93 (0.66–1.30)	1.20 (0.87–1.65)	1.74 (1.13–2.69)	0.010	
**Age**						0.214
<65	1.00	0.84 (0.59–1.20)	1.02 (0.69–1.52)	1.33 (0.81–2.19)	0.212	
≥65	1.00	1.21 (0.86–1.69)	1.47 (1.04–2.08)	1.50 (0.99–2.25)	0.020	
**Race**						0.428
White	1.00	1.08 (0.82–1.41)	1.26 (0.98–1.63)	1.49 (1.10–2.01)	0.005	
Black African American	1.00	0.85 (0.42–1.71)	1.58 (0.81–3.07)	1.52 (0.60–3.86)	0.233	
Other	1.00	0.43 (0.18–1.03)	0.73 (0.29–1.78)	0.80 (0.27–2.40)	0.588	
**Education**						0.384
High school graduate or below	1.00	0.95 (0.70–1.28)	0.98 (0.69–1.38)	1.01 (0.68–1.51)	0.909	
Some college/college graduate	1.00	0.89 (0.52–1.53)	1.53 (0.94–2.49)	1.66 (0.97–2.84)	0.025	
Post-college	1.00	1.13 (0.64–2.00)	1.26 (0.66–2.39)	2.41 (1.22–4.76)	0.038	
**Vigorous physical activity**						0.897
<2 times/week	1.00	0.99 (0.75–1.30)	1.14 (0.87–1.51)	1.33 (0.98–1.81)	0.046	
≥2 times/week	1.00	0.94 (0.53–1.67)	1.42 (0.77–2.62)	1.61 (0.80–3.23)	0.125	
**Smoking**						0.136
None	1.00	1.22 (0.84–1.77)	1.60 (1.16–2.21)	2.21 (1.34–3.64)	<0.001	
Ex-smoker	1.00	0.86 (0.61–1.21)	1.04 (0.73–1.48)	1.09 (0.73–1.62)	0.498	
Current smoker	1.00	0.39 (0.13–1.16)	0.39 (0.16–0.93)	0.41 (0.17–0.95)	0.185	
**Alcohol consumption**						0.108
No	1.00	0.78 (0.53–1.14)	1.24 (0.89–1.74)	1.40 (0.94–2.09)	0.017	
Yes	1.00	1.20 (0.89–1.62)	1.19 (0.83–1.70)	1.45 (0.96–2.18)	0.119	

Models adjusted for age, sex, race, education, energy intake, smoking, alcohol consumption, and physical activity. Stratification variables were not adjusted in the corresponding models.

## Data Availability

The dataset used in this study is based on a public open access repository released by the Health and Retirement Study (HRS): https://hrs.isr.umich.edu.

## Supplementary Materials

The following supporting information can be downloaded at https://www.mdpi.com/article/10.3390/nu16060760/s1, Figure S1: Factor loadings of dietary patterns among participants in the Health and Retirement Study; Table S1: Sample characteristics by Prudent Pattern quartiles among adults participating in the Health and Retirement Study; Table S2: Sample characteristics by Western Pattern quartiles among adults participating in the Health and Retirement Study.
