# Supplementary material for: Circadian Syndrome Is Associated with Dietary Patterns among Middle-Older Americans: The Health and Retirement Study
Source: Nutrients. 2024 Mar 7;16(6):760. doi: 10.3390/nu16060760 (PMC10975158; doi:10.3390/nu16060760)
Supplement: Supplementary file 1 [file nutrients-16-00760-s001.zip › nutrients-2860753-supplementary.pdf]

Supplemental Figure S1 Factor loadings of dietary patterns among participants attending Health and Retirement Study

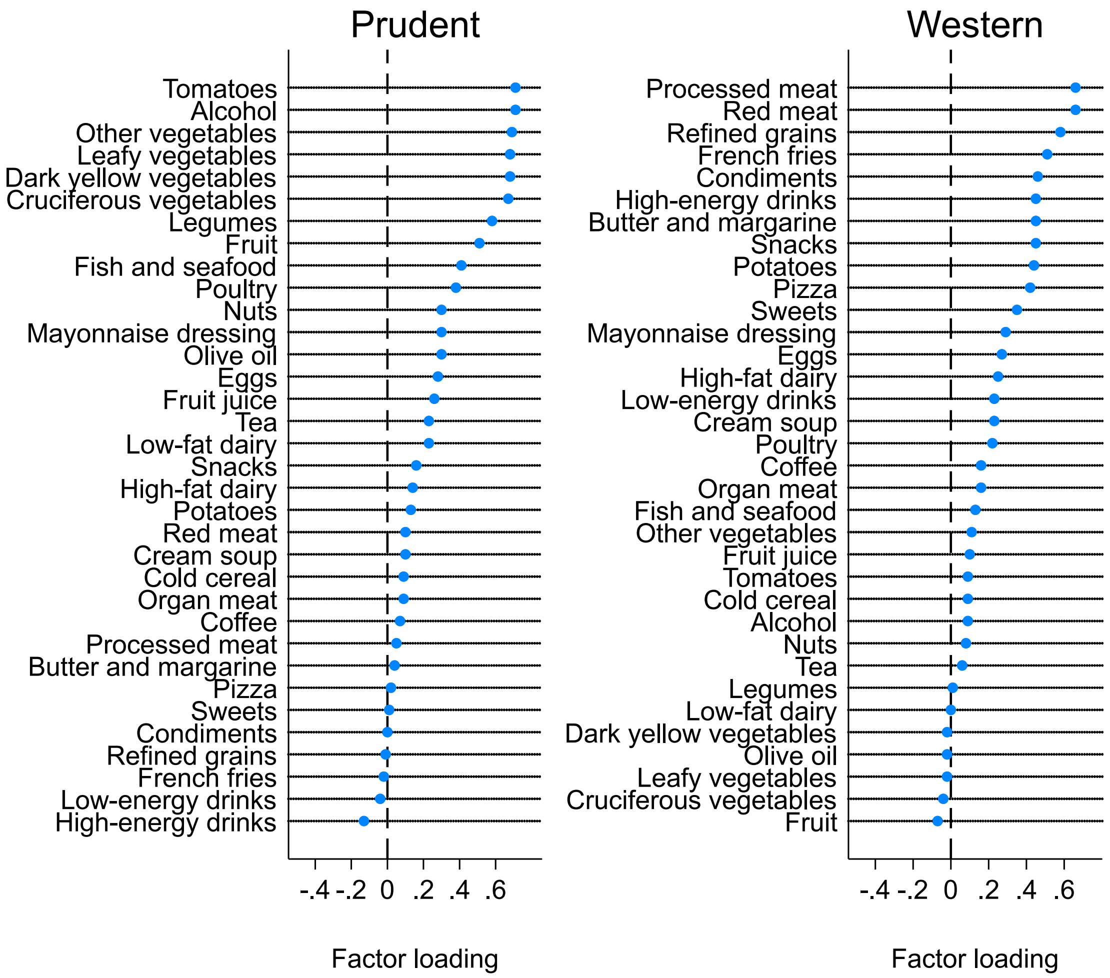

Variance explained (%): Prudent Pattern (14.0%), Western Pattern (8.6%).

Supplemental Table S1: Sample characteristics by quartiles of Prudent Pattern among adults attending Health and Retirement Study

	Total	Q1	Q2	Q3	Q4	p-value
	**N=4,253**	**N=1,064**	**N=1,063**	**N=1,063**	**N=1,063**	
Energy intake (kcal/day)	1830.0 (793.5)	1370.3 (640.2)	1605.6 (568.3)	1892.2 (633.6)	2452.2 (855.9)	<0.001
Protein intake (g/day)	71.9 (33.2)	50.6 (23.2)	62.4 (22.1)	75.4 (27.2)	99.1 (36.9)	<0.001
Fat intake (g/day)	67.5 (32.1)	50.0 (25.8)	59.2 (24.2)	70.6 (26.5)	90.4 (35.6)	<0.001
Carbohydrate intake (g/day)	229.7 (109.0)	173.1 (88.0)	200.4 (78.5)	234.4 (89.6)	310.9 (122.0)	<0.001
Western pattern	0.00 (1.00)	-1.00 (0.20)	-0.48 (0.15)	0.11 (0.20)	1.37 (0.88)	<0.001
Prudent pattern	-0.00 (1.00)	0.01 (0.99)	-0.05 (0.88)	0.02 (0.96)	0.02 (1.16)	0.24
Age (years)	65.4 (10.0)	64.8 (9.5)	66.2 (10.1)	66.0 (10.1)	64.7 (10.2)	<0.001
Gender						<0.001
Men	1,736 (40.8%)	488 (45.9%)	459 (43.2%)	404 (38.0%)	385 (36.2%)	
Women	2,517 (59.2%)	576 (54.1%)	604 (56.8%)	659 (62.0%)	678 (63.8%)	
Race						<0.001
White	3,286 (77.5%)	773 (72.8%)	834 (78.6%)	872 (82.2%)	807 (76.3%)	
Black African American	632 (14.9%)	214 (20.2%)	149 (14.0%)	133 (12.5%)	136 (12.9%)	
Other	323 (7.6%)	75 (7.1%)	78 (7.4%)	56 (5.3%)	114 (10.8%)	
Education						<0.001
High school grad or below	2,073 (48.8%)	644 (60.6%)	508 (47.9%)	449 (42.2%)	472 (44.4%)	
Some college/college grad	1,085 (25.5%)	260 (24.5%)	271 (25.5%)	287 (27.0%)	267 (25.1%)	
Post-college	1,092 (25.7%)	159 (15.0%)	282 (26.6%)	327 (30.8%)	324 (30.5%)	
Smoking						<0.001
None	1,934 (45.7%)	454 (43.0%)	468 (44.2%)	506 (47.8%)	506 (47.7%)	
Ex-smoker	1,889 (44.6%)	436 (41.3%)	495 (46.8%)	482 (45.5%)	476 (44.9%)	
Current smoker	410 (9.7%)	166 (15.7%)	95 (9.0%)	71 (6.7%)	78 (7.4%)	
Alcohol drinking						<0.001
No	1,838 (43.3%)	527 (49.6%)	469 (44.2%)	387 (36.4%)	455 (42.8%)	
Yes	2,411 (56.7%)	536 (50.4%)	592 (55.8%)	676 (63.6%)	607 (57.2%)	
**Vigorous physical activity**						<0.001
< 2 times/week	3,183 (75.2%)	887 (83.8%)	804 (76.1%)	791 (74.6%)	701 (66.4%)	
≥ 2 times/week	1,047 (24.8%)	172 (16.2%)	252 (23.9%)	269 (25.4%)	354 (33.6%)	
Central obesity	2,849 (71.6%)	747 (75.4%)	708 (71.2%)	716 (71.7%)	678 (68.3%)	0.006
Elevated glucose	2,129 (50.3%)	542 (51.2%)	514 (48.6%)	552 (52.1%)	521 (49.3%)	0.33
Elevated triglycerides	1,504 (35.4%)	418 (39.3%)	341 (32.1%)	375 (35.3%)	370 (34.8%)	0.006
Reduced HDL-C	1,059 (24.9%)	307 (28.9%)	256 (24.1%)	243 (22.9%)	253 (23.8%)	0.006
Elevated blood pressure	2,966 (69.8%)	781 (73.4%)	753 (70.9%)	723 (68.0%)	709 (66.7%)	0.003
Depression	543 (12.8%)	176 (16.5%)	127 (12.0%)	100 (9.4%)	140 (13.2%)	<0.001
Sleep disorder	1,345 (31.6%)	382 (35.9%)	352 (33.1%)	293 (27.6%)	318 (29.9%)	<0.001
Metabolic syndrome	2,055 (48.3%)	558 (52.4%)	483 (45.4%)	531 (50.0%)	483 (45.4%)	0.001
Circadian syndrome	1,527 (35.9%)	429 (40.3%)	363 (34.1%)	365 (34.3%)	370 (34.8%)	0.007

Data are presented as mean (SD) for continuous measures, and n (%) for categorical measures. *P-value < 0.05

Supplemental Table S2: Sample characteristics by quartiles of Western Pattern among adults attending Health and Retirement Study

	Total	Q1	Q2	Q3	Q4	p-value
	**N=4,253**	**N=1,064**	**N=1,063**	**N=1,063**	**N=1,063**	
Energy intake (kcal/day)	1830.0 (793.5)	1348.0 (560.1)	1542.2 (554.6)	1867.2 (584.3)	2562.8 (838.5)	<0.001
Protein intake (g/day)	71.9 (33.2)	54.2 (26.7)	62.0 (24.8)	73.7 (28.0)	97.5 (35.2)	<0.001
Fat intake (g/day)	67.5 (32.1)	46.4 (21.5)	56.0 (21.3)	69.0 (22.9)	98.7 (33.7)	<0.001
Carbohydrate intake (g/day)	229.7 (109.0)	178.5 (83.5)	193.0 (82.6)	232.9 (87.3)	314.4 (122.2)	<0.001
Western pattern	0.00 (1.00)	0.11 (1.09)	-0.09 (0.95)	-0.05 (0.91)	0.03 (1.03)	<0.001
Prudent pattern	-0.00 (1.00)	-1.02 (0.25)	-0.45 (0.14)	0.10 (0.19)	1.37 (0.87)	<0.001
Age (years)	65.4 (10.0)	65.7 (10.1)	65.6 (9.9)	65.8 (9.9)	64.6 (10.0)	0.027
Gender						<0.001
Men	1,736 (40.8%)	332 (31.2%)	394 (37.1%)	459 (43.2%)	551 (51.8%)	
Women	2,517 (59.2%)	732 (68.8%)	669 (62.9%)	604 (56.8%)	512 (48.2%)	
Race						<0.001
White	3,286 (77.5%)	774 (73.0%)	851 (80.4%)	855 (80.6%)	806 (76.0%)	
Black African American	632 (14.9%)	166 (15.7%)	137 (12.9%)	137 (12.9%)	192 (18.1%)	
Other	323 (7.6%)	120 (11.3%)	71 (6.7%)	69 (6.5%)	63 (5.9%)	
Education						<0.001
High school grad or below	2,073 (48.8%)	497 (46.8%)	493 (46.5%)	499 (46.9%)	584 (54.9%)	
Some college/college grad	1,085 (25.5%)	265 (24.9%)	257 (24.2%)	291 (27.4%)	272 (25.6%)	
Post-college	1,092 (25.7%)	301 (28.3%)	311 (29.3%)	273 (25.7%)	207 (19.5%)	
Smoking						<0.001
None	1,934 (45.7%)	565 (53.3%)	494 (46.6%)	488 (46.1%)	387 (36.7%)	
Ex-smoker	1,889 (44.6%)	441 (41.6%)	488 (46.0%)	481 (45.5%)	479 (45.4%)	
Current smoker	410 (9.7%)	54 (5.1%)	78 (7.4%)	89 (8.4%)	189 (17.9%)	
Alcohol drinking						0.053
No	1,838 (43.3%)	467 (44.0%)	437 (41.1%)	441 (41.6%)	493 (46.4%)	
Yes	2,411 (56.7%)	595 (56.0%)	626 (58.9%)	620 (58.4%)	570 (53.6%)	
**Vigorous physical activity**						<0.001
< 2 times/week	3,183 (75.2%)	734 (69.6%)	785 (74.3%)	799 (75.4%)	865 (81.7%)	
≥ 2 times/week	1,047 (24.8%)	321 (30.4%)	272 (25.7%)	260 (24.6%)	194 (18.3%)	
Central obesity	2,849 (71.6%)	660 (67.6%)	706 (70.5%)	730 (72.9%)	753 (75.6%)	<0.001
Elevated glucose	2,129 (50.3%)	524 (49.6%)	514 (48.5%)	527 (49.9%)	564 (53.2%)	0.17
Elevated triglycerides	1,504 (35.4%)	337 (31.7%)	347 (32.6%)	398 (37.4%)	422 (39.7%)	<0.001
Reduced HDL-C	1,059 (24.9%)	227 (21.3%)	239 (22.5%)	269 (25.3%)	324 (30.5%)	<0.001
Elevated blood pressure	2,966 (69.8%)	740 (69.6%)	694 (65.3%)	749 (70.5%)	783 (73.7%)	<0.001
Depression	543 (12.8%)	132 (12.4%)	125 (11.8%)	114 (10.7%)	172 (16.2%)	0.001
Sleep disorder	1,345 (31.6%)	316 (29.7%)	329 (31.0%)	334 (31.4%)	366 (34.5%)	0.11
Metabolic syndrome	2,055 (48.3%)	486 (45.7%)	467 (43.9%)	526 (49.5%)	576 (54.2%)	<0.001
Circadian syndrome	1,527 (35.9%)	342 (32.1%)	357 (33.6%)	391 (36.8%)	437 (41.1%)	<0.001

Data are presented as mean (SD) for continuous measures, and n (%) for categorical measures.*P-value < 0.05
